# Effect of Elemental Powder Size on Foaming Behavior of NiTi Alloy Made by Combustion Synthesis

**DOI:** 10.3390/ma5071267

**Published:** 2012-07-23

**Authors:** Yuya Arakawa, Makoto Kobashi, Naoyuki Kanetake

**Affiliations:** Graduate School of Engineering, Nagoya University, 1 Furo-cho, Chikusa-ku, Nagoya, Aichi 4648603, Japan; E-Mails: kobashi@numse.nagoya-u.ac.jp (M.K.); kanetake@numse.nagoya-u.ac.jp (N.K.)

**Keywords:** intermetallic foam, porous material, combustion synthesis, nickel titanium, foaming agent

## Abstract

Nickel titanium (NiTi) foams were made by combustion synthesis of powders with the help of ZrH_2_ as foaming agent and TiB_2_ as endothermic agent. In this paper, we investigated the effect of elemental powder size on the foaming. The powder size of Ni and Ti affected the ignition temperature of the combustion reaction, cell morphology and microstructure of the foams. The cell morphology of the foams was also modified by the powder size of TiB_2_.

## 1. Introduction

Various processes for fabricating porous NiTi alloys have been investigated by many researchers previously [1,2,3,4]. However, the processes available so far need large amount of energy to make porous materials because of a high melting point of the NiTi alloys. Our research group suggested a foaming process using combustion synthesis with the addition of an exothermic agent for AlTi porous materials [5,6]. This process reduces the amount of energy required to fabricate porous materials with high melting points by utilizing the heat that is produced during combustion reaction. We reported the foaming behavior of NiTi alloys made by this process and found that in addition to an endothermic agent (TiB_2_), a foaming agent was necessary to make such foams [7]. The heat of NiTi formation reaction is so high that the viscosity of molten NiTi during the reaction is very low. The endothermic agent addition decreases the highest temperature of the reaction and, therefore, increases the viscosity of molten NiTi. Cell wall rupture and pore coarsening in the NiTi foam during the combustion foaming are decreased by increasing the viscosity of the melt.

Besides, the elemental powder size affects the cell morphology of AlTi foams made by the combustion foaming process [6,8]. However, the effect of Ni, Ti and TiB_2_ powder size on combustion foaming process of NiTi is not known. Therefore we investigated that in this study. 

## 2. Experimental Procedure 

Nickel, titanium, an endothermic agent (titanium boride, TiB_2_) and a foaming agent powder (zirconium hydride, ZrH_2_) were used in this study. The sizes of these powders are given in Table 1. The molar blending ratio between Ni/Ti was fixed to 50.6/49.4 [9]. 32.5 vol% TiB_2_ and 0.5mass% ZrH_2_ were added in the NiTi mixture. Cylindrical compacts (φ = 15 mm, h = 15 mm, relative density = 0.7) were produced by cold pressing of the powder blend. The compacting pressure was 165 MPa.

**Table 1:** Nominal powder sizes as specified by the suppliers.

Powder	Denomination	Particle size (μm)	Purity (%)
Ni	Fine	3–5	99.9
	Intermediate	< 45	99
	Coarse	< 150	99.9
Ti	Fine	< 45	99.9
	Intermediate	90–150	99.9
	Coarse	150–250	99
TiB_2_	Fine	2–3	99
	Coarse	< 20	99
ZrH_2_	–	5	98

The compact was placed inside a crucible and ignited in a chamber filled with argon gas. A thermocouple was inserted in the powder compact to measure its temperature during the combustion reaction [7].

Porosity and pore diameter of the samples were measured from the cross sectional image by using image analyzing software [5,7]. The samples were characterized by X-ray diffractometer (XRD), scanning electron microscope (SEM) and energy dispersive X-ray spectrometer (EDS).

## 3. Results and Discussion

### 3.1. Effect of Powder Size on Temperature Profile of Combustion Synthesis

**Figure 1:**
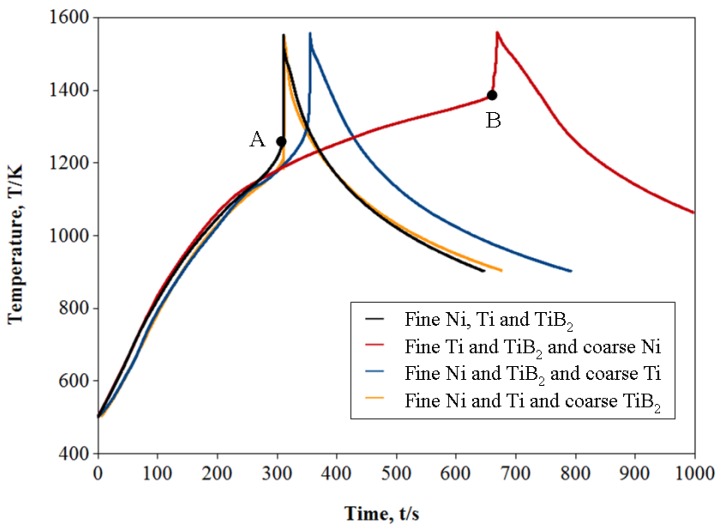
Temperature profile of the specimens made with various powder sizes of Ni, Ti and TiB_2_.

Figure 1 shows the temperature profiles of representative samples during the combustion reaction. The sharp increase in temperature indicates the ignition of the combustion reaction. The maximum temperature is referred to as combustion temperature, and it was reached near the melting point of NiTi alloy (1583 K). The ignition temperature of the combustion reaction was increased with increasing Ni or Ti powder size. This is due to the increased contacting surface area between Ti and Ni by using the finer elemental powders is regarded as a reason for the decrease of the ignition temperature. The same effect was previously reported by Thiers et al., but in the NiAl system [10]. The effect of Ni powder size was more remarkable than that of Ti powder size. This result is consistent with the work of Corbin *et al*., who discussed that the amount of intermetallic compound developed during sintering of Ni and Ti powder mixture was a strong function of the Ni particle size [11]. According [11], Ti phase was no longer observed in the micro structure of Ni/Ti powder compact made from coarse Ni and Ti powders, whereas Ni phase remained in the same sample after cooling from 1293 K. This means that the Ni phase is less reactive than the Ti phase below the ignition temperature of NiTi combustion reaction. In our experiments the use of fine Ni powder admixed with fine Ti and TiB_2_ powders resulted in a reduction of the ignition temperature by −150 K (from A to B) in comparison to the use of coarse Ni powder (as well admixed to fine Ti and TiB_2_) powders as can be seen in Figure 1. No significant change was observed in the temperature profile by changing the powder size of endothermic agent. 

### 3.2. Effect of Powder Size on Pore Morphology of Synthesized Foam

**Figure 2:**
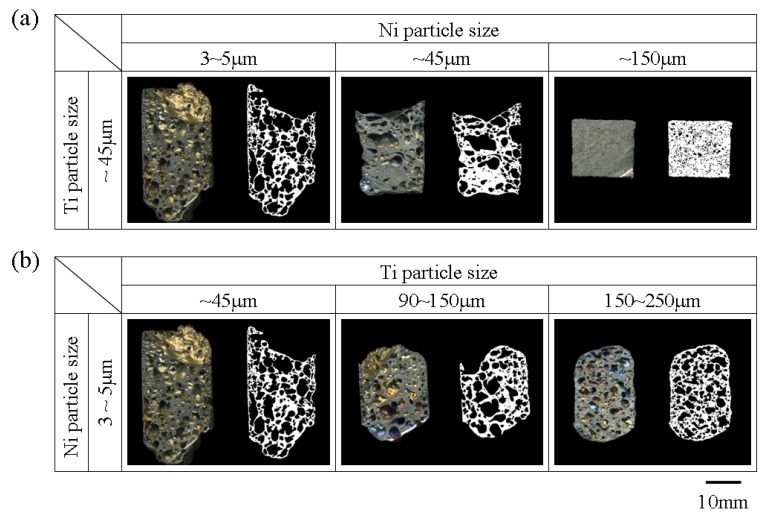
Cross section and binary image of combustion synthesized foams made using different (**a**) Ni; and (**b**) Ti particle sizes. TiB_2_ denomination is for both fine.

Figure 2 shows the cross section image and binary image of the foams with various powder sizes of Ni and Ti for the endothermic agent of fine size. Average porosities of the samples are shown in Table 2, Table 3. Each result is the average value of the measurement conducted on three different samples. The porosity and pore diameter of foams decreased by increasing the powder size of Ni and Ti. This occurs because the use of coarse Ni and Ti powders increased the ignition temperature but the foaming agent (ZrH_2_) releases H_2_ gas strongly above 773 K [12]. Therefore the higher the ignition temperature, the more H_2_ gas is lost before the combustion foaming starts. 

**Table 2:** Porosity of combustion synthesized samples using different size Ni powder.

Ni denomination	Particle size (μm)	Porosity (%)
Fine	3–5	60.00
Intermediate	< 45	42.10
Coarse	< 150	14.46
Ti denomination: fine (< 45 μm); ΤiB_2_ denomination: fine (< 2–3 μm)		

**Table 3:** Porosity of combustion synthesized samples using different size Ti powder.

Ti denomination	Particle size (μm)	Porosity (%)
Fine	< 45	60.00
Intermediate	90–150	47.34
Coarse	150–250	42.62
Ni denomination: fine (3–5 μm); ΤiB_2_ denomination: fine (< 2–3 μm)		

**Figure 3:**
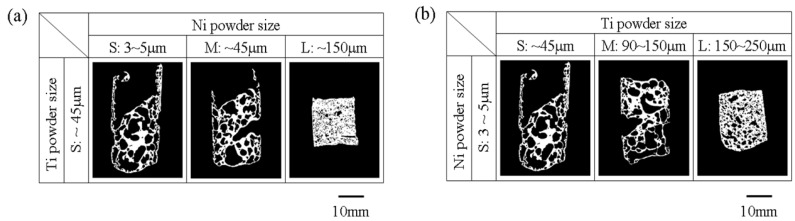
Combustion synthesized foams made using coarse TiB_2_ powder admixed with different (**a**) Ni; and (**b**) Ti particle sizes.

Figure 3 shows the binary image of the samples with various powder sizes of Ni and Ti but only coarse endothermic agent (TiB_2_). Fractured at outer cell wall and nonspherical shaped pores existed in relatively high porosity samples with fine Ti and fine Ni, fine Ti and intermediate Ni and intermediate Ti and fine Ni powders.

**Figure 4:**
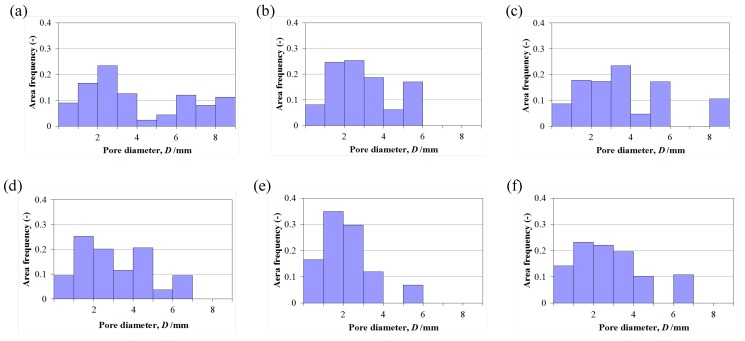
Histograms of pore diameter of samples with various powder sizes. (**a**–**c**) TiB_2_ powder denomination: coarse; (**d**–**f**) TiB_2_ powder denomination: fine; (**a**,**d**) Ni powder denomination: fine, and Ti powder denomination: fine; (**b**,**e**) Ni powder denomination: intermediate, and Ti powder denomination: fine; (**c**,**f**) Ni powder denomination: fine, and Ti powder denomination: intermediate.

Figure 4a–c shows the histogram of area frequency as a function of pore diameter for the sample sections shown in Figure 3 made with coarse TiB_2_ powder. Figure 4d–f are the same representations but for samples prepared with fine endothermic agent shown in Figure 2, *i.e.*, fine TiB_2_. These values are the sum of three samples. Figure 4 shows that the use of fine TiB_2_ has the positive effect of reducing the pore size of foams. This effect is more obvious in the high porosity foams. Thus the finer endothermic agent not only reduces the pore size but also stabilizes NiTi foams by preventing the collapse of outer cells probably because the finer solid TiB_2_ particles can stabilize better the foam in the liquid state. Previous studies support the observation that the addition of suitable solid particles increases the stability of metal foams and the stability increases by reducing the size of solid particles [13]. From these results, the fine endothermic agent (TiB_2_) powder is effective not only for absorbing the heat generated during the combustion reaction but also for stabilizing liquid NiTi foams in the liquid stage of combustion foaming.

### 3.3. Effect of Powder Size on Microstructure of Synthesized Specimen

**Figure 5:**
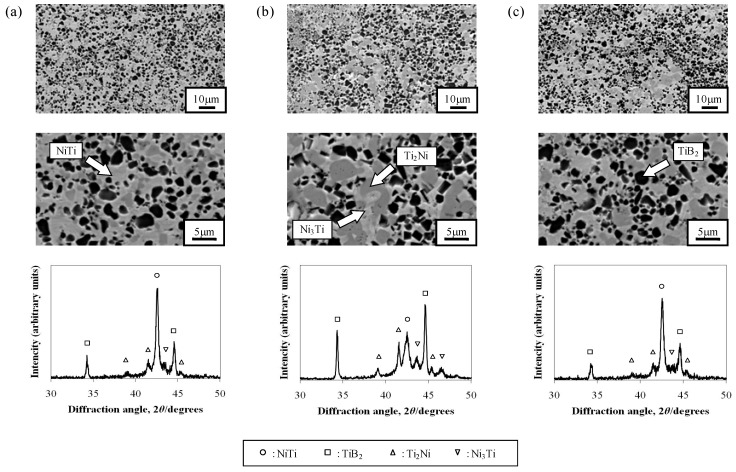
Micro structure and XRD spectra of combustion synthesized samples. (**a**) Ni powder denomination: fine, and Ti powder denomination: fine; (**b**) Ni powder denomination: coarse, and Ti powder denomination: fine; (**c**) Ni powder denomination: fine, and Ti powder denomination: coarse.

Figure 5 shows SEM images, EDS analysis and XRD spectra of samples with various powder sizes of Ni and Ti. Intermediate products of NiTi alloy (Ti_2_Ni and Ni_3_Ti) appear in the microstructure of all samples. However, the amount of these intermediate products in the sample made with coarse Ni and fine Ti powders was larger than that of the sample made by fine Ni and fine Ti powders. The larger amount of intermediate products on the sample prepared with coarse Ni powder occurred because the reaction to form NiTi is more incomplete due to the lower reactivity of Ni phase. As the Ti phase has a higher reactivity than Ni phase, the effect of Ti powder size on the micro structure is less remarkable.

From these results, it seemed that applying homogenization treatment by heating after the combustion foaming or using finer elemental powders than the elemental powders used in this work were needed to eliminate the intermediate products from the final products.

## 4. Conclusions 

The effect of Ti, Ni and TiB_2_ powder size on foaming behavior of NiTi alloy made by combustion synthesis was investigated: 

(1) Ignition temperature of combustion reaction of the samples increases with increasing powder size of both Ni and Ti, but the effect of Ni is more pronounced. In this regard, no significant change in the temperature profile was observed by changing the powder size of endothermic agent.

(2) Porosity and pore diameter of samples decrease by increasing the powder size of Ni and Ti. The effect of Ni powder size is also more remarkable because the use of coarse powders, especially coarse Ni powder, increases the ignition temperature above gas emission temperature of the foaming agent thus producing gas losses before foaming starts. Fractured outer cell walls and nonspherical shaped pores, and large pore diameter can be prevented by using finer endothermic agent TiB_2_. The reason for that is that solid fine TiB_2_ particles stabilize more effectively than coarse ones NiTi foams in the liquid state during combustion foaming.

(3) The intermediate products Ti_2_Ni and Ni_3_Ti appear in the microstructure of all samples. However, the amount of these non-desirable intermediate products is reduced by using fine Ni powder which helps complete the reaction to form NiTi. 
